# Interval Skin Necrosis in the Infrapopliteal Segment after Successful Distal Bypass Grafting in a Patient with Chronic Limb-Threatening Ischemia Complicated by Rheumatoid Arthritis Receiving Long-Term Corticosteroid Therapy

**DOI:** 10.3400/avd.cr.25-00071

**Published:** 2025-10-21

**Authors:** Tsutomu Doita, Shinsuke Kikuchi, Yuya Tamaru, Hirofumi Jinno, Keisuke Kamada, Naoya Kuriyama, Keisuke Miyake, Shigeru Miyagawa, Nobuyoshi Azuma

**Affiliations:** 1 Department of Vascular Surgery, Asahikawa Medical University, Asahikawa, Hokkaido, Japan; 2 Department of Chronic Limb-Threatening Ischemia, Asahikawa Medical University, Asahikawa, Hokkaido, Japan; 3 Department of Cardiovascular Surgery, Osaka University Graduate School of Medicine, Suita, Osaka, Japan

**Keywords:** chronic limb-threatening ischemia, interval ischemia, surgical wound complication

## Abstract

A 76-year-old woman with rheumatoid arthritis receiving long-term corticosteroid therapy, who underwent bilateral femoro-inframalleolar bypasses, suffered from interval skin necrosis in both lower legs after vein harvest in the contralateral leg and hematoma formation in the ipsilateral leg. Bilateral interval skin necrosis was improved eventually after revascularization for femoropopliteal lesions. In patients receiving long-term corticosteroid therapy who undergo distal bypass surgery, it is essential to address not only foot ischemia but also ischemia in the infrapopliteal region along the graft route and at the vein harvest site when formulating the surgical strategy.

## Introduction

Chronic limb-threatening ischemia (CLTI) often necessitates open bypass to manage limb perfusion. Despite a successful open bypass, the area proximal to the distal anastomosis can remain ischemic, leading to interval skin necrosis.^1)^ Although distal bypass surgery effectively restores perfusion to the foot in patients with CLTI, interval skin necrosis may still occur in the infrapopliteal segment along the graft route or at the vein-harvest incision site. This complication is particularly relevant in patients on long-term corticosteroid therapy, in whom skin fragility and small-vessel disease are common.^1)^ Herein, we report a case of serious interval skin necrosis after successful bilateral femoro-inframalleolar bypass in a patient with rheumatoid arthritis (RA) receiving prolonged corticosteroid therapy. This case required additional revascularization for residual femoropopliteal (FP) artery disease with severe calcification. The graft outcome, which was complicated by skin necrosis for 3 months, is also described in this report.

## Case Report

**Fig. 1:**
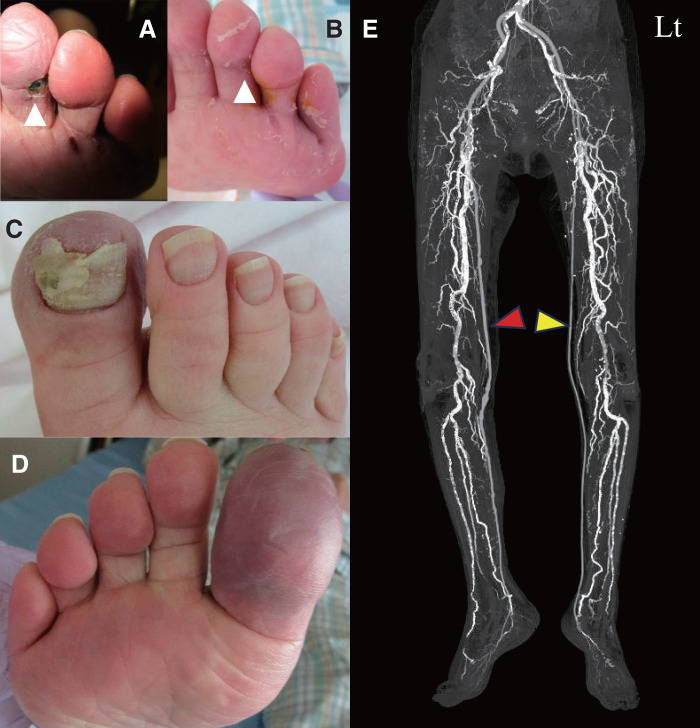
Bilateral foot before bypass surgery and CTA after bilateral bypass surgery. Left foot ischemic ulcer (**A**). Healed left foot ischemic ulcer after bypass surgery (**B**). Right cyanotic dorsal foot (**C**). Right cyanotic plantar foot (**D**). CTA images obtained following bypass surgery showing patent bilateral bypass grafts (red arrowhead, right; yellow arrowhead, left; **E**). CTA: computed tomography angiography; Lt: left

A 76-year-old woman with RA, who had undergone left deep femoral artery (DFA)–medial plantar artery bypass with a spliced vein graft using the bilateral great saphenous veins (GSVs) for CLTI with a second toe ulcer on the left foot a year ago, was referred to our department because of rest pain and cyanosis in the right foot. (**Figs. 1C** and **1D**). She had been diagnosed with RA 16 years earlier and had been receiving corticosteroid therapy continuously since then. At the time of presentation, RA was well controlled with oral corticosteroids (6 mg/day). Skin perfusion pressure in the right foot was 8 mmHg on both the dorsal and plantar surfaces, consistent with grade 3 ischemia according to the global vascular guidelines.^2)^ She was diagnosed with right CLTI. During the left-sided surgery, angiography had demonstrated left FP lesions consisting of focal occlusions with severe calcification, classified as FP grade 3. The left medial plantar artery was supplied by collateral flow, and the left distal bypass to the medial plantar artery was deemed sufficient to treat the left CLTI. Computed tomography angiography (CTA) after the left distal bypass revealed that the right leg had lesions similar to those on the left. Therefore, a right distal bypass was planned in the same manner as the previous left-sided procedure. The patient lacked adequate GSV because of the previous procedure; thus, a right superficial femoral artery (SFA)–medial plantar artery bypass was performed using a spliced vein graft composed of the left small saphenous vein (SSV) and the left cephalic vein on hospital day 8. The procedure was successful, and postoperative CTA and ultrasound sonography demonstrated a patent bypass graft, and her rest pain and cyanosis in the right limb improved (**Fig. 1E**).

**Fig. 2:**
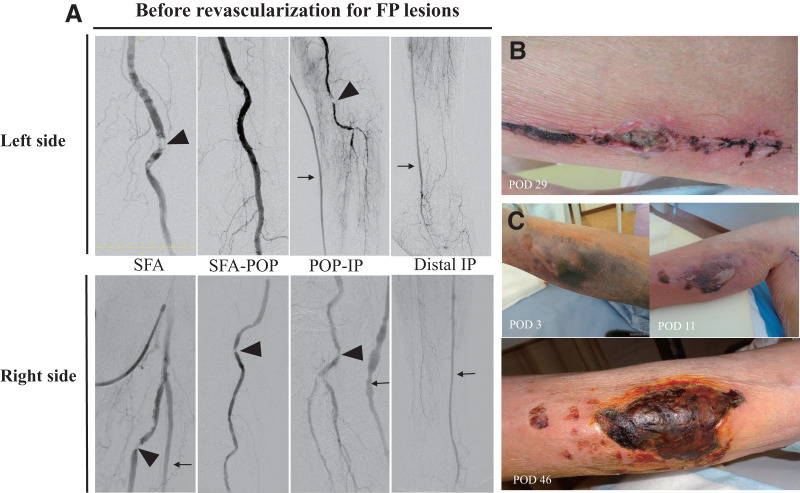
Preoperative angiography and postoperative findings: bilateral FP lesions, left SSV harvest site, and right thigh hematomas resulting from graft tunneling. Severe stenosis is observed in the left proximal SFA and the below-knee segment of the popliteal artery (arrowheads), along with advanced disease in the infrapopliteal arteries. Retrograde blood flow from the left bypass graft (arrow) is poor due to the severity of the infrapopliteal lesions. On the right side, severe stenosis affects both the proximal and distal segments of the SFA, as well as the popliteal artery (arrowheads). Similarly, retrograde flow from the right bypass graft (arrow) is compromised by severe infrapopliteal disease (**A**). Wound dehiscence at the left SSV harvest site, observed 29 days after right-sided bypass surgery (**B**). A subcutaneous hematoma developed postoperatively, gradually darkened, and progressed to necrosis (**C**). FP: femoropopliteal; SFA: superficial femoral artery; POP: popliteal artery; IP: infrapopliteal artery; POD: postoperative day; SSV: small saphenous vein

On postoperative day (POD) 29, the SSV harvest site on the left lower leg showed delayed wound healing (**Fig. 2B**). Moreover, the right lower leg, where the graft was placed after tunneling, exhibited a change in skin color to black with necrotic status on POD 46 (**Fig. 2C**). Based on the angiography findings of both legs, we suspected ischemic skin lesions and measured tissue oxygen saturation (StO_2_) using a tissue oximeter OXY-2 (ViOptix, Fre-mont, CA, USA). StO_2_ in the left lower leg ranged roughly from 6% to 19%, which was lower than 32% at the left ankle and 31% at the left thigh, indicating that the delayed wound healing was associated with lower limb ischemia. The right side was also assessed as ischemic skin necrosis.

**Fig. 3:**
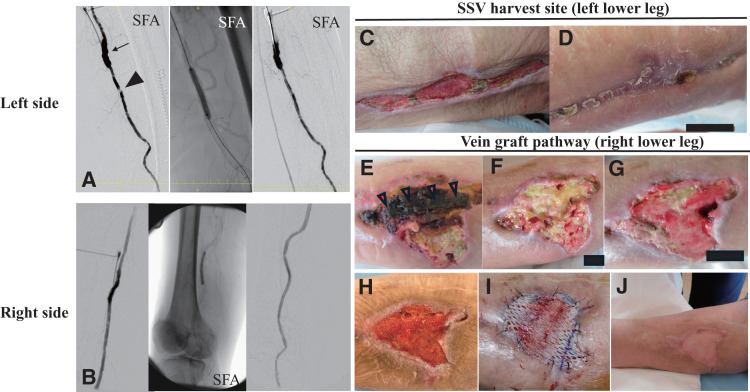
Following femoropopliteal revascularization, bilateral lower extremity angiography revealed residual lesions and postoperative wound changes. On the left side, after bovine patch angioplasty of the proximal SFA, a residual lesion was identified and treated with balloon angioplasty (**A**). On the right side, bovine patch angioplasty was performed for the proximal SFA, and the distal SFA was subsequently revascularized by balloon angioplasty (**B**). Twenty-three days after the left hybrid procedure, the SSV harvest site showed signs of delayed healing (**C**). By postoperative day 79, the wound had completely healed without further intervention (**D**). On the right side, a subcutaneous hematoma developed over the bypass graft, which was located just beneath the necrotic area (arrowheads), as observed on postoperative day 37 (**E**). By day 44, the necrotic skin had been completely removed (**F**). The wound continued to evolve over time, with notable changes documented on days 58 (**G**), 87 (**H**), and 105, at which point the wound was covered with a split-thickness skin graft (**I**). Complete wound healing was achieved by day 120 following the right hybrid procedure (**J**). SFA: superficial femoral artery; SSV: small saphenous vein

Endovascular therapy was considered because of focal arterial lesions; however, the bilateral SFA lesions were occluded with severe calcifications. Since the endovascular approach could cause distal embolization and subsequent acute limb ischemia in both lower legs, we decided on hybrid revascularization: endarterectomy and patch angioplasty with bovine pericardium (XenoSure; LeMaitre, Burlington, MA, USA) for the SFA lesions, and balloon angioplasty for the distal popliteal artery lesions. On the left side, the hybrid procedure was performed 42 days after the bypass surgery. The popliteal artery lesions shown in **Fig. 2C** were difficult to cross with a guidewire due to severe calcification, and thus the lesion was not treated because the inflow reconstruction was sufficient to perfuse collateral flow to the vein harvest site (**Fig. 3A**). Subsequently, the same revascularization was performed on the right side 63 days after the bypass surgery (**Fig. 3B**).

Postoperative findings showed that StO_2_ in the left lower leg improved to 30%–56%, and StO_2_ around the right skin necrosis improved to 28%–62%. At 79 days after the left hybrid procedure, the left SSV harvest site healed with negative pressure wound therapy (NPWT) and maintenance debridement (**Figs. 3C** and **3D**). The black necrotic skin on the right side gradually formed a scab, which was carefully removed because the bypass graft was located just beneath (**Fig. 3E**). After complete removal, NPWT and maintenance debridement were performed (**Figs. 3H**–**3J**). Finally, complete wound healing was achieved by skin grafting at 120 days after the hybrid procedure (**Figs. 3I** and **3J**). The patient’s hospital stay was extended to 191 days. Thirty-seven days after the completion of right wound healing, the right vein graft exhibited only focal stenosis above the knee, not in the area of the lower leg skin necrosis. This stenotic lesion was managed with plain old balloon angioplasty on 2 occasions over a period of 2 years. Remarkably, both vein grafts have remained patent for over 2 years. The patient has maintained her ambulatory status and has been free of ulcers for the same duration.

## Discussion

This case report highlights the occurrence of interval skin ischemia in the lower leg due to FP arterial lesions following distal bypass surgery for CLTI. This complication is relatively rare, and there are a limited number of publications addressing this specific clinical issue. The significance of such wound complications has received only minor emphasis, and these troublesome cases are not often reported in the literature.^3)^ This case involves a patient with RA undergoing long-term corticosteroid therapy. It is presumed that the significant skin fragility and small vessel disease in this patient have greatly contributed to the complication. Additionally, these patients often face immune dysfunction and are frequently administered steroids, which further complicate wound healing. The combination of immune dysfunction, corticosteroid use, and small vessel disease collectively increases the risk of surgical site infections and complicates postoperative recovery.^4)^

In this case, skin necrosis was exacerbated by ischemia in the lower leg, in addition to the patient’s inherent skin fragility. Typically, ischemia in this region is not a major concern in bypass surgery; however, the presence of severe FP lesions and small vessel disease, combined with skin fragility, resulted in significant lower leg ischemia. This ischemia was assessed using StO_2_ measurements. Normally, the lower leg exhibits StO_2_ values of approximately 60%–70% at rest, which can drop to around 30% during exercise in patients without lower extremity arterial disease (LEAD), and to about 10% in those with LEAD.^5)^ In this case, the patient’s lower leg StO_2_ was already critically low at 10%–20% at rest. Revascularization of the FP lesions improved StO_2_ values to approximately 30%–60%, facilitating healing. The distal lower leg showed StO_2_ values of about 30% due to retrograde perfusion from the distal bypass, indicating that interval ischemia in the lower leg was a significant clinical issue in this case. Considering reports that the closure and disruption of the DFA and geniculate collateral pathways pose a high risk for the development of interval ischemia,^6)^ a retrospective evaluation of this case’s angiography reveals extensive calcification throughout the entire region. Furthermore, the poor development of the DFA and geniculate collateral pathways around the knee likely contributed to the occurrence of interval ischemia.

Poor wound healing can significantly affect the prognosis of vein grafts. Studies have shown that inadequate wound healing is associated with higher rates of graft failure and complications.^7)^ Traditionally, treatment has included graft excision and relocation. For vein grafts, aggressive local treatment of infected lower extremity bypass grafts, including drainage, debridement, and muscle transposition, may treat infection in selected patients without the need for graft removal and with rates of limb salvage superior to those obtained with excisional therapy.^7)^ In the current case, graft relocation was not performed since additional skin incisions could lead to delayed wound healing. Fortunately, the vein graft did not become infected and has maintained patency for 2 years with minor interventions. Ensuring optimal wound care and monitoring is crucial for improving graft patency and overall patient outcomes. Given these considerations, it is imperative to provide meticulous care for surgical wounds, especially in patients receiving long-term corticosteroid therapy. When performing infrainguinal bypass in such patients, it is advisable to position the bypass graft under the fascia. This approach helps mitigate the risk of delayed wound healing, which could otherwise threaten the integrity of the graft.

## Conclusion

Long distal bypass in cases with severe SFA or popliteal lesions can result in interval skin necrosis due to residual ischemia, leading to wound complications that threaten the graft. In patients with CLTI receiving long-term corticosteroid therapy, it is crucial to address not only the distal target but also FP lesions and collateral development to effectively manage vein graft-related wounds.
